# American Veterinary College

**Published:** 1897-05

**Authors:** 


### AMERICAN VETERINARY COLLEGE.

THE annual meeting of the Alumni Association of the American Veterinary College was held in the College Hall at 3 P.M., March 25, 1897. Among those present were: Drs. W. H. Lowe, H. D. Hanson, F. R. Hanson, W. B. E. Miller, W. H. Pendry, L. H. Howard, W. Horace Hoskins, Herbert Neher, Otto Faust, C. L. Adams, H. W. Bieser, A. H. Blake, W. J. Coates, E. F. Hogan, E. H. Ackerman, M. W. Drake, M. J. Murphy, R. W. Ellis, L. D. Ives, E. N. Leavy, E. L. Volgenau, F. R. Ogden, Hermann Koch, W. C. Brotherton, and Prof. Olof Schwarzkopf, of Chicago, Ill.

After the reading and adoption of the minutes of the meeting of 1896 the Executive Committee made their report, showing provision for the annual dinner and alumni prize and the consideration of the necessary alterations of the constitution and by-laws, among others a recommendation to establish a list of those members of the Association not in good standing who declined to pay the annual fee of $1. The election of officers resulted in the selection of Dr. L. H. Howard, President; H. D. Hanson, First Vice-President;

R. W. Ellis, Second Vice-President; Otto Faust, Secretary; F. R. Hanson, Treasurer; Herman Bieser, Librarian. The deaths reported were of Drs. Autenreith, Peralta, Leich, and Southwick, and committees were appointed by the Chair to draw up suitable resolutions in recognition of their death and the loss to the Association.

President Howard's address was carefully prepared and added much to the history of the A. V. C. boys as supporters and earnest workers in the upholding and advancement of the veterinary profession. He referred to the many positions held by them of an official character, and of the thoroughness with which much of the work of the profession was done by members of our Alumni Association. The meeting adjourned at a late hour with a cordial invitation to all the members of the Association to attend the commencement exercises, which were held in Chickering Hall at 8 P.M.

The following programme covered the exercises:

Overture, "Gallathea," (Suppe.) Selection, "Geisha," (Jones.) March, "Valerose," (Wittig.) Prayer, Rev. Adolphus T. Sieker. Intermezzo, "Naila," (Delibes.) Conferring of Degrees, Faneuil D. Weisse, M.D., President Board of Trustees. Selection, "Lady Slavey," (Kerker.) Awarding of Prizes, Prof. L. H. Friedburg, Ph.D. Waltz, "Life's Lullaby," (Bucalossi.) Valedictory, George Bass Blackman, D.V.S., of the graduating class. Selection, "Popular Airs," (Beyer.) Address, Rev. Lindsey Parker, D.D. Benediction, Rev. Adolphus T. Sieker. March, "El Capitan," (Sousa.)

The following students received the Degree of Doctor of Veterinary Surgery: George Bass Blackman, Harry Wentworth Bellman, Jr., George De Witt Boice, Horace Winfield Boyd, Arthur Launy Grover, Bernard Gunther, Howard Suydam Holdenby, James Grant Hope, M.D.C., Ralph Collyer Jenks, John Victor Laddey, Otto Rudolph Leis, James Edward Masterson, John Phillip Messer, John Joseph Murray, Thomas John Ogle, John Gregory Slee, Harry Balthasar Shugar, Thomas Francis Smith, George Stephens, Franklin Smith, C. Chauncey Tietjens, John Thomas, George Frederick Westcott.

Rev. Lindsay Parker delivered the address, and it was one of the most appropriate, interesting, and humorous extemporaneous addresses ever given to a fully appreciative audience. It was interspersed with wholesome advice, and many brilliant thoughts flashed rapidly from the speaker, which fell upon willing ears and minds to grasp and utilize the same.

The valedictory by George Bass Blackman was a thoughtfully prepared one, giving an adequate idea of the work of the veterinarian; and his remarks to the faculty were happily made, with a thorough appreciation of the sincere efforts of their labors and in their behalf. His parting words to his fellow-graduates, while fully appreciating and willingly accepting the responsibilities that must fall to their lot, were hopeful in that the outgoing class might shed as much honor and lustre over their alma mater as those which had preceded them. The music was of a varied character, very enjoyable, and added much to the pleasure of the occasion.

At the close of the exercises the Faculty, trustees, and graduates repaired to the Hotel Marlborough, where was arranged a series of decorated tables, around which groups of those attending spread themselves, and enjoyed, until an early hour of the morning, the wealth of good things provided to satisfy the inner man, and listened to many suggestive and pointed remarks from members of the trustees, faculty, and graduates, drawn forth by the persuasive powers of the toastmaster, Dr. William H. Lowe. Among those who responded to the toasts were Dr. F. D. Weisse, of the Board of Trustees; Professors Robertson, Coates, and Hanson, of the Faculty; many of the older graduates and member of the graduating class, including the class historian, thus fittingly closing the twenty-second annual reunion of the Alumni Association.

